# Circular Approach to Biomanufacturing: Enhancing Therapeutic Protein Production Using Chum Salmon Head Peptone

**DOI:** 10.3390/bioengineering13040409

**Published:** 2026-03-31

**Authors:** Svini Dileepa Marasinghe, Minthari Sakethanika Bandara, Somyong Lee, Young Hwa Kim, Su-Jin Lee, Dong Soo Hwang, Youngdeuk Lee, Eunyoung Jo, Tae-Yang Eom, Gun-Hoo Park, Chulhong Oh

**Affiliations:** 1 Jeju Bio Research Center, Korea Institute of Ocean Science and Technology, 2670, Iljudong-ro, Gujwa-eup, Jeju 63349, Republic of Korea; 2 Department of Marine Technology & Convergence Engineering (Marine Biotechnology), University of Science and Technology, 217, Gajeong-ro, Yuseong-gu, Daejeon 34113, Republic of Korea; 3 Graduate School of Ferrous & Eco Materials Technology, Pohang University of Science and Technology (POSTECH), Pohang 37673, Republic of Korea; 4 Inflamm-Aging Translational Research Center, Ajou University Medical Center, Suwon 16499, Republic of Korea

**Keywords:** *Oncorhynchus keta*, chum salmon head peptone (CSHP), recombinant protein, *Escherichia coli* BL21(DE3), expression, life cycle assessment (LCA)

## Abstract

Fish waste disposal poses significant environmental and economic challenges, limiting sustainability in the marine food industry. Hence, sustainable valorization strategies are needed to enhance resource recovery while minimizing waste. As an approach, this study aimed to evaluate the potential of converting chum salmon (*Oncorhynchus keta*) head (CSH) waste into a high-value peptone for microbiological applications. Various proteolytic enzymes were screened for CSH hydrolysis, among which Protamex achieved the highest hydrolysis and recovery rates. The resulting chum salmon head peptone (CSHP) exhibited favorable characteristics, including a low average molecular mass (557 Da) and a high amino nitrogen content (4.9%), outperforming commercial animal (AP) and vegetable (VP) peptones. To assess its biotechnological potential, CSHP was evaluated as a nitrogen source for recombinant protein production and supported higher expression of human superoxide dismutase (hSOD) and human growth hormone (hGH) in *Escherichia coli* BL21(DE3), compared with AP, VP, and Luria–Bertani (LB) media. Furthermore, life cycle assessment revealed a substantially lower carbon footprint for CSHP production than that of conventional peptone sources. These findings suggest that CSHP is a reliable and sustainable alternative to traditional peptones, offering both therapeutic and industrial applications while contributing to marine waste reduction and circular bioeconomy strategies.

## 1. Introduction

Globally, the seafood industry is pivotal in ensuring food security and economic stability, but faces the challenge of managing substantial volumes of waste generated annually [1]. It is estimated that 30–70% of the fish harvested in fisheries and aquaculture becomes waste or by-products, most of which arises during fish processing [2]. These by-products, including heads, bones, skin, scales, fins, flesh, and viscera, are often discarded or underutilized, contributing to huge economic losses and environmental concerns [3]. Inadequate measures taken to address the use or disposal of these by-products result in pollution and greenhouse gas emissions. Thus, innovative strategies are required to be urgently formulated for repurposing fish waste into valuable resources [4].

In the fish processing industry, a considerable portion of by-products is frequently wasted, with the estimated quantity ranging from 20% to 80% of the total fish weight [5]. However, protein-rich side streams can be effectively recovered and utilized through various processes such as enzymatic hydrolysis, chemical hydrolysis, and bacterial fermentation [6]. As the by-products constitute a high percentage of the total weight of fish, measures need to be taken to utilise these materials to their full potential. Fish by-products, which are rich in proteins, lipids, and bioactive compounds, make them promising raw materials for the development of high-value products, including biofertilizers, animal feed, nutraceuticals, pharmaceuticals, and cosmeceuticals [7]. Among these applications, the development of peptones represents a particularly valuable strategy. Peptones are water-soluble protein hydrolysates widely used in microbiology, biotechnology, and pharmaceutical industries [8]. They play a crucial role in microbial growth and support the cultivation of recombinant microorganisms that produce therapeutic proteins [9]. Despite the fact that research on the production of peptone using fish by-products has been conducted across various fish species, the majority of this research mainly focused on enhancing the microbial growth [10,11,12,13,14]. However, a recent study compared the productivity of recombinant proteins produced using peptone derived from cutlassfish heads with that of commercially available peptones, suggesting better performance in recombinant protein expression [15]. Furthermore, the antioxidant and anti-inflammatory properties of the cutlassfish head peptone have also been demonstrated [16].

Despite these advances, the need persists for the identification and development of new environmentally responsible peptone sources that align with local fishery by-product streams and robust growth-supporting properties. Hence, the research efforts were directed toward the development of peptones from Chum salmon (*Oncorhynchus keta*), a species widely distributed in the North Pacific Ocean, with approximately 76% of the global catch being sourced from the Russian, Japanese, and Korean waters of the northwestern Pacific [17]. Global production of chum salmon has been estimated to be approximately 200,000–300,000 tonnes annually, with variability influenced by environmental fluctuations and stock dynamics [18]. During processing, about 40–60% of the fish biomass becomes by-products, representing a substantial source of protein-rich materials for value-added applications. Chum salmon, in particular, is recognized for its pharmaceutical value, with its skin gelatin aiding in wound healing [19], whereas roe extracts provide antioxidant and anti-aging benefits for skin health [20]. Chum salmon heads (CSH) make up a considerable fraction of the total fish weight. Hence, their underutilization significantly contributes to the waste management challenges while representing a missed economic opportunity. Therefore, using CSH for peptone production may fulfil the dual purpose of mitigating waste disposal issues and creating a value-added ingredient that is crucial for biotechnological processes.

In this context, establishing sustainable production systems is essential to support the increasing demand for recombinant proteins in therapeutic and industrial sectors. Human superoxide dismutase (hSOD) and human growth hormone (hGH) are two such proteins with significant biomedical and industrial applications. hSOD is a critical antioxidant enzyme that catalyses the dismutation of superoxide radicals into oxygen and hydrogen peroxide to protect cells from oxidative damage and maintain cellular homeostasis [21]. Owing to its potent antioxidative properties, hSOD is a suitable candidate for therapeutic applications in neurodegenerative diseases, inflammatory conditions, and cosmetic formulations aimed at anti-aging and skin protection [22]. hGH is a peptide hormone that is crucial for regulating cell growth, metabolism, and tissue regeneration [23,24]. It is widely used as a therapeutic to treat growth hormone deficiencies and muscle-wasting diseases and as a performance-enhancing agent in sports and regenerative medicine [24,25]. The industrial production of these proteins relies heavily on recombinant DNA technology, with *Escherichia coli* serving as a preferred expression system owing to its rapid growth, cost-effectiveness, and high protein yield [26].

The aim of the study was to address the critical issue of marine waste reduction by converting CSH into a novel, high-quality bioactive peptone. Chum salmon head peptone (CSHP) was evaluated as a potential nitrogen source for enhancing the recombinant production of hSOD and hGH in *E. coli*. The specific objectives were threefold: (1) to develop and optimise an efficient protein hydrolysis method for the production of CSHP; (2) to evaluate and compare the capacity of CSHP to enhance the recombinant protein production of hSOD and hGH in *E. coli*, relative to commercial peptones; (3) to assess and compare the carbon footprint of the CSHP production process with that of conventional media components. The rationale was to improve the protein yield in industrial applications while simultaneously harnessing the reduced carbon footprint and resource circularity associated with the use of local fish by-products, as this could be a significant step toward increasing sustainability in bioprocessing practices [27]. Through this approach, we aimed to enhance the overall value of the fisheries and aquaculture industry. We believe that the findings of this study on transforming a commonly deemed waste into a valuable biotechnological resource would contribute to sustainable resource management and support advancements in therapeutic protein manufacturing and other industrial applications.

## 2. Materials and Methods

### 2.1. Raw Material Preparation

Chum salmon head samples (10.4 kg, wet weight) were supplied by Organ Eco Tech company (Seongnam-si, Republic of Korea). The heads were washed with tap water and steamed at 100 °C for 30 min. Then, the muscle tissues, bones, and remaining contents were collected separately. The CSH muscles were freeze-dried and ground into a fine powder.

### 2.2. Peptone Production Using Chum Salmon Head Muscle

#### 2.2.1. Optimization of Protein Hydrolysis

The hydrolysis efficiency of salmon heads was optimized using a variety of commercially available proteases, namely, bromelain (Sigma-Aldrich, Burlington, MA, USA), papain (Sigma-Aldrich, Burlington, MA, USA), pronase (Roche, Basel, Switzerland), Protamex (Sigma-Aldrich, Burlington, MA, USA), and trypsin (Gibco, Life technologies, Carlsbad, CA, USA), which were used according to the optimal pH conditions indicated in Table S1. Preliminary experiments were performed using CSH muscle powder to determine the optimal enzyme quantity required for the hydrolysis process. CSH muscle powder was mixed with distilled water, followed by pH adjustment to the optimal range with 2N NaOH. Hydrolysis reactions were carried out at a substrate concentration of 1% (*w*/*v*), with enzyme addition at 1% (*w*/*v*), which corresponds to enzyme activities in each reaction mixture of >30 U/mL (bromelain); 15–100 U/mL (papain); 70 U/mL (pronase); >0.015 AU-N/mL (Protamex); and >2500 USP units/mL (trypsin)**,** for 6, 12, 18, and 24 h at 45 and 55 °C with agitation at 150 rpm. The reaction was deactivated after the specified time interval by heating the sample mixtures at 95 °C for 10 min. The samples were centrifuged at 8000 rpm for 10 min to separate the supernatant containing the hydrolyzed soluble fraction and the pellet containing the unhydrolyzed insoluble fraction. The supernatant was subjected to sodium dodecyl sulfate-polyacrylamide gel electrophoresis (SDS-PAGE) to identify the bands of the hydrolyzed proteins. The pellets were oven-dried at 60 °C until a constant mass was observed and used to evaluate the recovery rates of the samples digested using each enzyme under various conditions. The recovery rate was calculated as follows:

Recovery rate (%) = (Mass of the recovered product/Initial substrate mass) × 100(1)

where the mass of the recovered product was obtained by the reduction of the final mass of the pellet from the initial substrate mass.

#### 2.2.2. Peptone Production from Chum Salmon Head Muscle

Protamex was used to produce CSHP. Briefly, CSH muscle powder (5% *w*/*v*) was mixed with distilled water, and the pH was adjusted to 7. Then, 0.6% (*w*/*v*) Protamex was added, and the reaction mixture was incubated at 55 °C for 6 h to induce hydrolysis. Next, the mixture was subjected to heating at 95 °C for 10 min to inactivate the enzyme. The sample was centrifuged at 10,000 rpm for 20 min. The upper soluble layer of the mixture was collected and freeze-dried to prepare the CSHP.

### 2.3. Chemical Characterization of Peptones

#### 2.3.1. Determination of Molecular Mass Distribution

Gel permeation chromatography (GPC) was employed to analyse the molecular mass distributions of CSHP, animal peptone (AP), and vegetable peptone (VP) (Sigma-Aldrich). Weight average molecular mass (Mm), number average molecular mass (Mn), Molecular mass of highest peak (Mp) and the polydispersity index (PDI) were measured using Waters e2695 instrument equipped with Ultrahydrogel columns (120, 250, 500, 1000; 7.8 mm × 300 mm, Waters, Milford, MA, USA) and a refractive index detector (RID). CSHP, AP, and VP (5 mg each) powders were separately dissolved in 1 mL of distilled water and filtered through a 0.45 µm membrane. At 35 °C, 100 µL aliquots were injected into the system. The mobile phase was 0.1 M sodium nitrate (NaNO_3_) with a flow rate of 1 mL/min. A calibration curve for the standard was generated by plotting the logarithm of the relative molecular mass (Mm, Da) of the standard versus its retention time (t, min), and the subsequent data analysis was performed.

#### 2.3.2. Determination of Total Nitrogen Content

The total nitrogen content of the CSHP was analyzed and compared with that of the AP and VP. Distilled water (80 mL) was added to a decomposition test tube, and 25 mL of a mixture of the indicator solution was poured into an Erlenmeyer flask, which was then placed inside the distillation apparatus. Then, 50 mL of 40% sodium hydroxide (NaOH) was added to the decomposition tube; this volume was equivalent to four times the sulfuric acid used for decomposition. Distillation was performed for 3–4 min, and the distillate was titrated with hydrochloric acid (HCl) solution until the appearance of a light pink colour, which indicates the endpoint. Total nitrogen content was calculated using the following equation:

Total nitrogen (%) = [(HCl consumed − Blank) × M × 14.0]/Sample weight(2)

where HCl consumed indicates the volume of HCl (mL) used in titration; Blank indicates the volume of HCl (mL) used in the blank test; M indicates the molar concentration of HCl; 14.01 indicates the atomic weight of nitrogen; and sample weight indicates the weight of the sample in mg.

#### 2.3.3. Determination of Amino Nitrogen Content

The amino nitrogen content of the CSHP, AP, and VP was analyzed using the Sorensen formal titration method. Briefly, 2 mL of the sample was diluted with 100 mL of distilled water, and the mixture was stirred for 1 h. The pH of the solution was initially adjusted to 8.4 using 0.2 N NaOH. Then, 20 mL of 35% formaldehyde was added, and the pH of the solution was readjusted to 8.4 using 0.2 N NaOH. A blank test was performed using distilled water separately to establish a baseline for the amino nitrogen content. The amino nitrogen content was calculated using the following equation:

Amino nitrogen (mg) = 2.8 × [VT − (VH + Ve)] × F(3)

where VT indicates the total volume (mL) of 0.2 N NaOH used up to the third stage; VH indicates the volume (mL) of 0.2 N NaOH equivalent to the added 0.2 N HCl solution; Ve indicates the total volume (mL) of 0.2 N NaOH added to the control solution; and F indicates the factor of the 0.2 N NaOH solution.

#### 2.3.4. Determination of Amino Acid Profiles

Constituent amino acid content was analyzed by measuring the quantity of the CSHP sample that was hydrolyzed using 30 mL of 6N HCl at 130 °C for 24 h. Next, the mixture was diluted up to 50 mL using ultrapure water and filtered using a 0.45-µm syringe filter. The hydrolyzed samples were further diluted with ultrapure water and analyzed using a high-pressure liquid chromatography (HPLC) system (Agilent 1200LC, Agilent Technologies Inc., Santa Clara, CA, USA) under the conditions described in Table S2.

Free amino acid content was analyzed by mixing 1 g of CSHP sample with 0.1% perchloric acid and 0.1 M meta-phosphoric acid. Then, triple-distilled water was added to the mixture to make up the volume to 50 mL. After dilution, the mixture was subjected to ultrasonic extraction (WUC-D22H, Daehan Scientific Co., Ltd., Gangwon-do, Republic of Korea) for 1 h. Further extraction was performed for 1 h at room temperature, and the supernatant was filtered through a 0.2-µm syringe filter. The collected samples were analyzed using an amino acid analyser (Dionex UHPLC3000 Systems, Dionex Corporation, Sunnyvale, CA, USA) under the conditions described in Table S2. The calibration curve for the quantitation of amino acids was plotted at standard concentrations of 10, 100, 500, and 1000 pmol/µL.

#### 2.3.5. Analysis of Heavy Metals and Histamine Levels

Heavy metal concentrations (Pb, Hg, As, and Cd) were determined by an accredited external analytical institution (Regional Innovation Center, Jeju National University, Republic of Korea) following the official methods described in the Korean Food Code. Histamine levels in the CSHP medium were also quantified using validated methods specified in the Korean Food Code. All analyses were conducted under standardized quality-controlled conditions.

### 2.4. Analysis of Peptone Efficiency in Recombinant Protein Production

#### 2.4.1. Cloning of Human Superoxide Dismutase and Human Growth Hormone Genes

pET-11a vectors containing separate *hSOD* and *hGH* genes were provided by the Korea Institute of Ocean Science and Technology (Jeju, Republic of Korea). The *hSOD* and *hGH* genes were genetically modified through codon optimization to include a chitosanase signal peptide originating from *Bacillus subtilis* strain CH2. The corresponding nucleotide sequences for *hSOD* and *hGH* were submitted to GenBank under accession numbers OR661283 and PQ766613, respectively. Primers were designed for *hSOD* and *hGH* to ensure the removal of the chitosanase signal peptide. The following forward and reverse primers were used: *hSOD* gene, 5′-ACGAAGGCAGTCTGC-3′ (5′ phosphorylation) and 5ʹ-CATATGTATATCTCCTTCTTAAAGT-3′ (5′ phosphorylation); *hGH* gene, 5′-TTTCCGACAATCCCC-3′ (5′ phosphorylation) and 5′-CATATGTATATCTCCTTCTTAAAGT-3′ (5′ phosphorylation), respectively. Polymerase chain reaction (PCR) was performed using a 50-µL reaction mixture containing 10 pmol each of the forward and reverse primers, 10–20 ng of template deoxyribonucleic acid (DNA), 4 µL of 2.5 mM deoxyribonucleotide triphosphate (dNTPs), 10 µL of 5x PrimeSTAR GXL buffer, and 1 µL of primeSTAR GXL DNA polymerase (Takara Bio Inc., Shiga, Kusatsu, Japan). The PCR cycle conditions were as follows: for *hSOD*, initial denaturation at 94 °C for 5 min; 30 cycles of denaturation at 98 °C for 10 s, annealing at 55 °C for 15 s, and extension at 72 °C for 6.2 min; and final extension at 72 °C for 5 min; for *hGH*, initial denaturation at 94 °C for 5 min; 30 cycles of denaturation at 98 °C for 10 s, annealing at 60 °C for 15 s, and extension at 68 °C for 6 min; and final elongation at 72 °C for 5 min. PCR product formation was confirmed on a 1% agarose gel, and the bands were purified using the Accuprep Gel Purification Kit (Bioneer, Daejeon, Republic of Korea). The purified PCR products were self-ligated using T4 DNA ligase (Takara Bio Inc., Shiga, Japan) and separately introduced into *E. coli* DH5α cells through heat shock transformation. The transformed cells were cultured overnight in Luria–Bertani (LB) broth containing ampicillin (100 μg/mL) at 37 °C and under shaking at 180 rpm. The cells were collected by centrifugation, and their plasmid DNA was extracted using the AccuPrep^®^ Plasmid Extraction Kit (Bioneer, Daejeon, Republic of Korea). The nucleotide sequences were confirmed by performing sequencing (Macrogen, Seoul, Republic of Korea). The plasmid DNA was transferred to the expression host *E. coli* BL21(DE3) via heat shock.

#### 2.4.2. Bacterial Cell Growth and Recombinant Protein Overexpression in Various Peptone-Based Media

To evaluate the effect of peptone source on the growth of *E. coli* BL21(DE3), media were formulated to resemble the standard LB composition (1% peptone, 0.5% yeast extract, and 1% NaCl) in which the peptone component was substituted with VP, AP, and CSHP. Overnight culture of *E. coli* BL21(DE3) was obtained by incubating 4 mL LB medium at 37 °C with shaking at 180 rpm. Volumes of 500 µL of overnight culture were transferred into 20 mL of each test medium, and the bacterial growth performance was monitored using a Real-Time Cell Growth Logger (Biosan, Riga, Latvia), which utilizes RTS tubes equipped with aeration membranes. Optical density (OD_850_) measurements were recorded at 10 min intervals over a 48 h period, and the resulting data was used for subsequent analysis. The experiment was further extended to evaluate the overexpression of recombinant proteins hSOD and hGH across different culture media. Each broth medium was formulated with the same base composition as standard LB broth (1% peptone, 0.5% yeast extract and 1% NaCl), with 1% peptone component replaced by the respective test peptone (VP, AP, and CSHP). The *E. coli* transformants containing the inserted genes were inoculated into 4 mL of the prepared LB media separately and cultured overnight at 37 °C. Next, 500 µL of the bacterial culture was transferred into 20 mL of culture medium containing different peptones. Protein expression was induced by adding 0.1 mM isopropyl-β-D-thiogalactopyranoside (IPTG) when the culture reached an optical density of 0.6–0.7 at 600 nm, followed by incubation at 20 °C for 24 h. Subsequently, the samples were collected and centrifuged for 1 min to obtain the cell pellets, which were disrupted by sonication, followed by analysis of the cellular proteins using SDS-PAGE. Protein quantification was performed using ImageJ 1.54g software (NIH, Bethesda, MD, USA), and bovine serum albumin (BSA; Thermo Fisher Scientific, Waltham, MA, USA) was used as the standard. The protein expression experiments were repeated thrice separately in different batches.

### 2.5. Life Cycle Assessment

**Figure 1:**
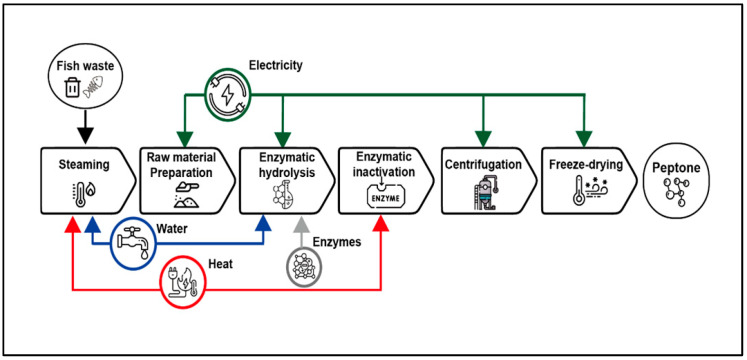
System boundary and processing steps for CSHP production used in the LCA. The process included steaming, raw material preparation (freeze-drying and grinding), enzymatic hydrolysis, enzyme inactivation, centrifugation, and freeze-drying. Operating parameters such as raw material input, temperature, residence time, pH, enzyme dosage, and yield were recorded for each step. These detailed values were used to normalize the life cycle inventory (LCI) to the functional unit of 1 kg peptone in the LCA.

The life cycle assessment (LCA) was performed to assess the environmental impact of peptone production from three raw materials: CSH, defatted soybean meal, and skimmed milk. Among these, chum salmon head–derived peptone (CSHP) was the main focus of this study. The workflow and system boundary for CSHP production are illustrated in Figure 1, and all mass and energy flows were normalized to the functional unit of 1 kg peptone to ensure comparability.

These materials were selected owing to their availability, varying environmental impacts, and relevance to both traditional and alternative production methods. Soybean and skimmed milk peptones were specifically chosen because they are among the most widely used representatives of vegetable-based and animal-based peptones in industrial microbiological and biotechnological applications [28,29,30]. Their frequent use in commercial culture media provides a robust benchmark for comparison with salmon head–derived peptone. For comparability, all three systems were modeled under a standardized workflow. In addition, process parameters reported in patents and literature [31,32,33] were used to recalculate soybean- and skimmed milk–based peptone production, with detailed inventory data provided in Table S3. For salmon head production, fish heads were treated as wastes entering the system under a burden-free cut-off assumption (ISO 14044). To ensure fairness, a sensitivity analysis was also conducted under a co-product allocation approach, with results reported in Table S4.

Environmental indicators such as greenhouse gas emissions (GWP100) were calculated using the ReCiPe Midpoint (H) method within the ecoinvent 3.9.1 database, and all inventories were integrated into OpenLCA (v1.11.0). The analysis focused on capturing the key differences in resource use and emissions across processes. For the LCA inventory, we assumed the same operating conditions as described in Section 2.2 for peptone production, while all inputs and outputs were normalized to the functional unit of 1 kg of peptone. However, due to the absence of specific datasets in ecoinvent, certain inputs (Protamex) were represented using functionally similar proxy datasets such as generic enzyme production. The complete mapping of substituted materials is provided in Table S5. This method provides a robust foundation for evaluating the environmental benefits of peptone production from salmon head compared to soybean and skimmed milk. Greenhouse gas emissions, expressed as the Global Warming Potential (GWP100) values derived during the LCA, were used to assess the environmental benefits of the different peptone production processes. Emission reduction was calculated as follows:

Absolute reduction in emissions was assessed based on the absolute reduction in greenhouse gas emissions between two processes, which was calculated using the following equation:

Reduction (absolute) = GWP of Reference Process − GWP of Alternative Process(4)

Percentage emission reduction was assessed based on the relative reduction (expressed as a percentage), which was computed using the following equation:

Reduction (%) = [GWP_reference − GWP_alternative)/GWP_reference] × 100(5)

### 2.6. Statistical Analysis

All statistical analyses were performed using GraphPad Prism (Version 9.5.1, GraphPad Software, San Diego, CA, USA). Differences among the recovery rates obtained for enzyme-treated samples and the recombinant protein concentrations in four different culture media were compared using two-way and one-way analysis of variance (ANOVA), respectively, followed by Tukey’s multiple comparison test as the post hoc test. Data are presented as the mean ± standard error of the mean (SEM).

## 3. Results and Discussion

### 3.1. Selection of Commercial Protease for Chum Salmon Head Muscle Hydrolysis

In the current study, we focused on extracting peptones from chum salmon fish waste specifically for biotechnological and pharmaceutical applications rather than converting them into regular animal feed. Among the various parts of the fish, the head region is a major processing by-product, generated in substantial quantities and containing a considerable amount of muscle protein. Fish heads, typically considered inedible, account for approximately 20% of the total fish weight [34]. For chum salmon, a total of 10.4 kg of fish heads were obtained, from which 2.25 kg (wet weight) of head muscle tissue was recovered, accounting for 21.6% of the total head weight (Table 1).

**Figure 2:**
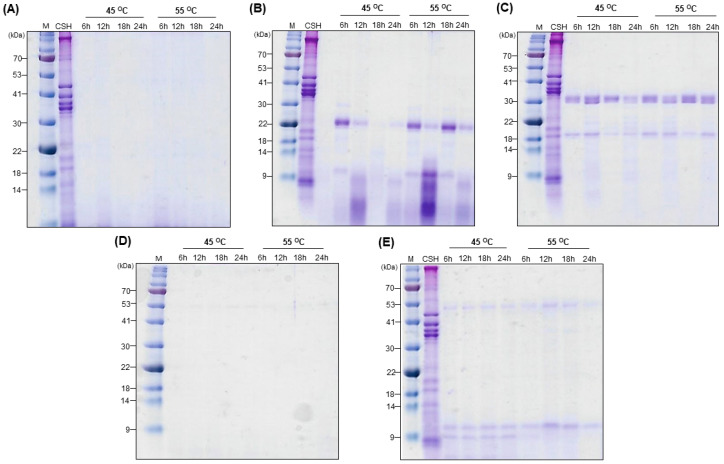
SDS-PAGE analysis of hydrolyzed CSH muscle fractions treated with different proteolytic enzymes. Five different enzymes, (**A**) bromelain, (**B**) papain, (**C**) pronase, (**D**) Protamex, and (**E**) trypsin, were used to hydrolyse CSH muscle fractions at 45 and 55 °C for 6, 12, 18, and 24 h. The lane abbreviations indicate the following: M, protein ladder; CSH, chum salmon head muscle proteins.

Proteins derived from plant or animal sources using chemical, enzymatic, or fermentation methods can be used to generate high-quality peptides that benefit living organisms [35]. Generally, polypeptide chains with molecular masses ≥ 8000 Da are classified as proteins [36], and the conversion of these high-molecular-mass proteins into low-molecular-mass peptides and amino acids through hydrolysis is commonly evaluated by the degree of hydrolysis. Although the degree of hydrolysis measures the percentage of peptide bonds cleaved, it does not directly reflect the molecular mass distribution of the resulting peptides, which is an important parameter in the peptone media development. Therefore, SDS-PAGE analysis was used to visualize the protein degradation by observing the reduction or disappearance of high-molecular mass protein bands. The protein degradation patterns observed in SDS-PAGE profiles provide insight into the peptide size distribution, which is an important factor in microbial culture media, as small peptide fragments and free amino acids are more readily assimilated during microbial growth. However, SDS-PAGE does not provide information related to the yield of the recovered hydrolysate. Therefore, recovery rate was evaluated, to reflect the extent of protein solubilization and the overall yield of the hydrolysate which represents amount of soluble nutrients available for microbial growth. Based on these considerations, both SDS-PAGE analysis and recovery rate were used as suitable indicators to evaluate the extent of hydrolysis. Figure 2 illustrates the SDS-PAGE analysis of CSH muscle hydrolysates generated using commercially available enzymes: bromelain, papain, pronase, Protamex, and trypsin at 45 °C and 55 °C under their respective optimal pH conditions. No bands were observed on SDS-PAGE for CSH muscle hydrolysates produced after bromelain and Protamex treatments regardless of the incubation time or temperature. This indicates the breakdown of high molecular mass protein bands into smaller peptide fragments and amino acids, which had likely migrated out of the gel owing to their reduced size (This interpretation is supported by GPC results, indicating that the Protamex–treated peptone is composed of low molecular-mass-peptides; see Table 2). Additionally, several protein bands with varying molecular masses were observed on SDS-PAGE for CSH muscle hydrolysates produced after papain, pronase, and trypsin treatments indicating incomplete protein hydrolysis.

**Figure 3:**
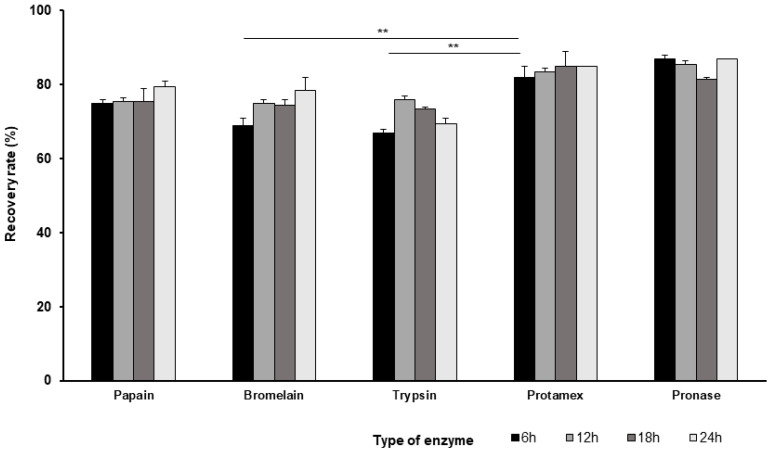
Effect of commercially available enzymes on the hydrolysis of CSH muscle. The muscle tissue was hydrolyzed using five different enzymes, bromelain, papain, pronase, Protamex, and trypsin, at 55 °C for time intervals of 6, 12, 18 and 24 h. Protamex-treated sample at 6 h intervals was compared with other enzyme-treated samples at 6 h using two-way ANOVA followed by Tukey’s multiple comparison test. Data are presented as mean ± SEM. Significant differences are indicated as follows: *p* < 0.01 (**).

Figure 3 demonstrates the recovery rates of the hydrolyzed CSH muscles measured after hydrolysis for different time intervals (6, 12, 18, and 24 h) at 55 °C. All enzymes exhibited good performance, and the recovery rates exceeded 65% for all time intervals. The recovery rate provides an indication of the extent of transformation of the insoluble protein into a water-soluble form, which helps assess the effectiveness of the treatment. However, this method does not directly quantify the extent of protein hydrolysis. The recovery rates for each enzyme at the shortest (6 h) and longest (24 h) time intervals were: bromelain, 69% and 78%; papain, 75% and 79.5%; pronase, 87% and 87%; Protamex, 82% and 85%; and trypsin, 67% and 69.5%. Among the enzymes, Protamex and pronase exhibited effective hydrolysing ability with recovery rates above 80% for all the tested time intervals. Statistical analysis revealed no significant difference in recovery rates across 6, 12, 18, and 24 h time intervals for both Protamex and pronase, indicating that the shortest incubation period (6 h) is sufficient for effective hydrolysis. While both Protamex and pronase proved highly effective in hydrolysing CSH muscles, the detection of residual protein bands at 30 and 18 kDa in pronase-treated samples indicates incomplete fish protein degradation. Hence, only Protamex was considered suitable for CSH muscle hydrolysis. At the 6 h time point, the recovery rate of Protamex-treated samples was significantly different than that of bromelain- and trypsin-treated samples (*p* < 0.01), whereas no significant differences were observed when compared with papain and pronase. Considering both recovery yield and the protein degradation patterns, Protamex was identified as the most preferred enzyme under the tested conditions, producing a higher recovery yield with low-molecular-mass peptide fragments.

### 3.2. Chum Salmon Head Peptone Characterization

CSHP production involves the treatment of the CSH muscle with the enzyme Protamex. The obtained peptone was dissolved in water, and it did not exhibit any signs of aggregation upon heating. Through hydrolysis, proteins break down into shorter peptides and amino acids, thereby improving their solubility. Additionally, an increase in the number of polar and hydrophilic groups reduces the molecular size, which improves solubility [37]. Moreover, the molecular mass and size distributions of fish protein hydrolysates significantly influence their functional and bioactive properties [38]. Hence, GPC analysis was performed to determine the molecular mass distribution of the CSHP (Table 2). CSHP analysis revealed an average molecular mass of 557 Da, the lowest among the three peptones tested (AP, 642 Da; and VP, 1222 Da). This indicates that CSHP is composed of peptides with smaller molecular sizes than those present in AP and VP. The polydispersity index (PDI) of CSHP was 1.93, reflecting a broad range of molecular mass distribution. Furthermore, this PDI value was lower than those of VP (2.08) and AP (2.51). This trend could be attributed to the fact that the peptides produced after CSHP degradation are composed of smaller units.

The total nitrogen and amino nitrogen contents of AP and VP were analyzed and compared with those of the obtained CSHP (Table 3). AP showed the highest total nitrogen content of 15.2%, but had the lowest amino nitrogen content of 2.6%. This high nitrogen content of AP can be attributed to the presence of nitrogen from other sources in addition to peptide or amino acid-based nitrogen. In contrast, CSHP exhibited a lower total nitrogen content compared to AP, but it had the highest amino nitrogen content of 4.9%, suggesting a significant contribution from peptide- or amino acid-based nitrogen sources. However, VP exhibited lower total nitrogen (11.5%) and amino nitrogen (3.4%) levels compared to CSHP.

Furthermore, the amino acid composition of CSHP was analyzed as a part of its characterization. Since amino acids are the building blocks of proteins, evaluating their composition in peptones helps assess their potential in recombinant protein production. Analysis of the constituent amino acid profile of CSHP revealed that glutamic acid was the most abundant amino acid with a relative content of 17.9%, followed by aspartic acid (10.4%), lysine (8.83%), and leucine (8.17%) (Table S6). In contrast, the free amino acid profile showed that leucine (19.2%) was the most abundant, followed by taurine (11.6%), phenylalanine (8.01%) and lysine (7.16). Taurine is a β-amino acid and is identified as the second most abundant amino acid in the profile; it elicits a range of effects, including antioxidant, anti-inflammatory, and protein folding-enhancing properties and ER stress inhibition [39]. Unlike constituent amino acids, free amino acids represent the bioavailable fraction of nitrogen source, which can be readily absorbed by the cell for cellular metabolism and protein production [40]. During protein synthesis, amino acids are linked to form polypeptides via translation in the ribosome. Therefore, a sufficient supply of amino acids is essential for efficient protein synthesis. Consequently, the deficiency of specific amino acids can hinder the rate of polypeptide formation. Hence, the type and the abundance of amino acids available in the peptone directly influence the recombinant protein synthesis. In the current study, CSHP was utilized as a nitrogen source for producing human-related recombinant proteins (hSOD and hGH). Compositional analysis of hGH revealed that leucine accounts for 13.4% of its constituent amino acid content. The high free leucine content in CSHP (19.2%) suggests that it could support hGH production. Furthermore, efficient aminoacylation of tRNA (aminoacyl-tRNA charging) during protein translation is essential for proper ribosomal protein synthesis [41]. The findings of this study suggest that CSHP may enhance tRNA charging efficiency and contribute to the maintenance of translation speed.

The concentrations of heavy metals and the histamine level in CSHP were analyzed to ensure the safety for biotechnological applications and to minimize the possible health risks associated with the fish-based materials. The heavy metal analysis indicated the presence of trace amounts of cadmium (0.07 mg/kg) and mercury (0.02 mg/kg) in CSHP, whereas lead and arsenic were not detected in the developed peptone medium. When the detected levels of heavy metals increase, they can be mitigated through careful raw material selection, optimizing processing steps, and regular heavy metal monitoring during quality control of the final product [42]. Studies also highlighted the possible reduction of heavy metals, such as Hg levels in fish meals, through centrifugation and micro/nano filtration of tuna hydrolysates [43]. Moreover, the CHSP medium is free of histamines, indicating the minimization of potential adverse effects on industrial applications and ensuring safety in various biotechnological processes.

### 3.3. Bacterial Cell Growth and Recombinant Protein Production Using Chum Salmon Head Peptone Medium

**Figure 4:**
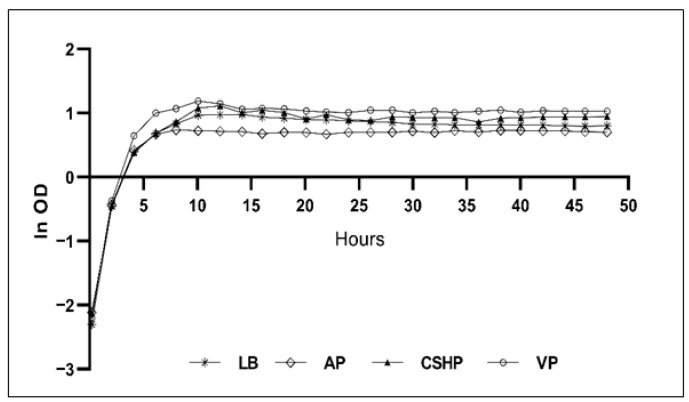
Cell growth profiles of *E. coli* BL21(DE3) across different peptone-based media (LB, AP, CSHP and VP). Each medium was inoculated with an overnight culture, and the bacterial growth was monitored by measuring optical density at 850 nm over 48 h.

*E. coli* is one of the most widely used microbes for the production of a vast array of products, including biochemicals [44], biopharmaceuticals [45], biomaterials [46], and biofuels [47] due to its well-characterized biology and easy genetic manipulation [48]. One of the major goals in recombinant protein expression is to produce large quantities of desired proteins through an expression host. However, protein expression may sometimes be complicated by low or non-production of the protein of interest [49]. Factors such as the choice of expression hosts, fusion tags, inducers, promoters, media composition, and process optimization need to be appropriately managed to obtain high-quality soluble proteins [45]. This study focuses on evaluating the impact of different culture media on *E. coli* cell growth and recombinant protein expression. Cell growth experiments with *E. coli* BL21(DE3) were performed using four different culture media formulated with VP, AP, CSHP and LB. Among the tested media, the medium containing VP supported the highest cell growth (Figure 4) followed by the CSHP medium, whereas the AP resulted in the lowest cell growth performance. A high bacterial growth rate can be attributed to the availability of nutrients in the culture media. It has been recognized that the uptake of various nutrients by bacteria is closely linked with their metabolic outcomes such as growth, energy production and biomass accumulation [50]. Selvarasu et al. have reported the effect of nutrient composition in complex media on the growth of *E. coli* DH5α cells and identified several nutrient components influencing the phenotypic behavior of cells. Among the amino acids, serine exhibited the highest uptake rate during the initial exponential growth phase. Moreover, the study revealed a sequential consumption order of serine, aspartate and glutamate from the complex medium [51]. An analysis of the free amino acid profiles of VP, AP, and CSHP, revealed the presence of highest proportions of serine, glutamate and aspartate in VP compared to AP and CSHP (Table S7), which may be a reason for better bacterial growth performance observed in VP medium. However, CSHP also supported in growth performance comparable to that achieved with standard LB media, demonstrating its potential as a competitive alternative.

**Figure 5:**
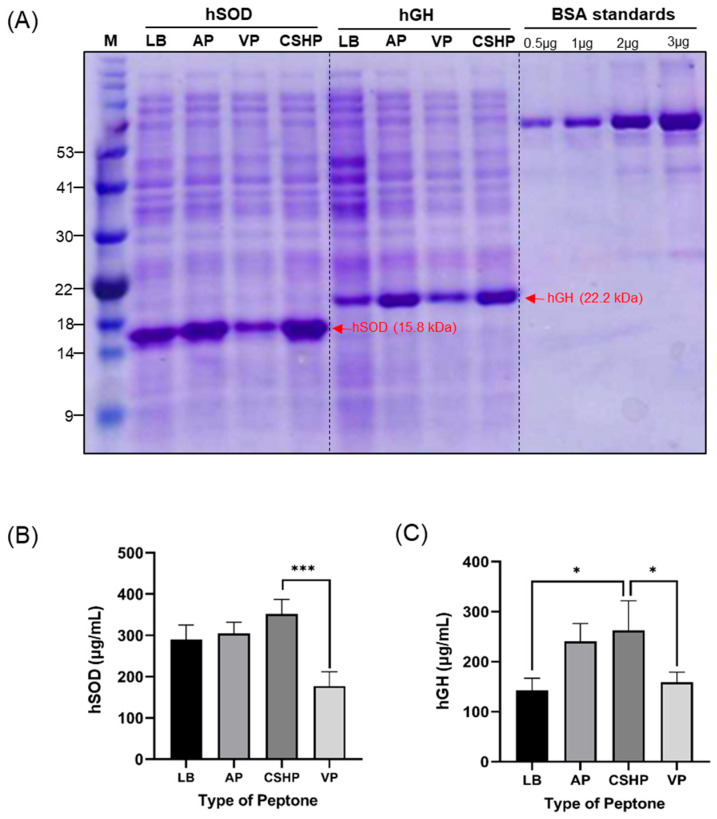
Expression of hSOD and hGH proteins in various peptone-containing culture media. (**A**) SDS-PAGE analysis of recombinant proteins. Lane abbreviations: M, protein marker; LB, Luria–Bertani; AP, animal peptone; VP, vegetable peptone; CSHP, chum salmon head peptone. Lanes with bovine serum albumin (BSA) at different concentrations of 0.5, 1, 2, and 3 µg were used as the standards to quantify protein expression. (**B**) Comparative analysis of hSOD protein expression in different media. (**C**) Comparative analysis of hGH protein expression in different media. The four groups were compared using one-Way ANOVA followed by Tukey’s multiple comparison test. Data are presented as mean ± SEM. Significant differences are indicated as follows: *p* < 0.05 (*), *p* < 0.001 (***).

The industrial production of human recombinant proteins incurs high initial costs at several stages but shows low production yields in most cases. Therefore, several techniques have been studied to enhance protein production. The expression of soluble proteins is greatly influenced by the components of the culture media, including salts, peptones, and yeast. Hence, understanding the relationship between the media constituents and soluble protein expression is vital for exploring novel and cost-effective materials such as fish waste for peptone production; therefore, we investigated the effect of the culture medium containing CSHP on the production of two types of human-related recombinant proteins: hSOD and hGH. hSOD, an antioxidant enzyme eliminating reactive oxygen species, plays a therapeutic role in neuro-protection, wound healing, anti-tumor activity and anti-aging applications [52], whereas hGH is used in treatment of growth disorders and exhibits tissue-regenerative, wound healing and anti-aging properties [53]. These proteins were expressed in LB medium, which is a standard medium for *E. coli* cultivation, and in three other media formulated using AP, VP, and CSHP. As shown in Figure 5, both CSHP and AP media showed enhanced protein yields compared to those of standard LB medium. Specifically, hSOD concentrations obtained using the CSHP and AP media were 352 ± 30 µg/mL and 304 ± 22 µg/mL, respectively, whereas those obtained using LB and VP media were 290 ± 28 µg/mL and 178 ± 26 µg/mL, respectively. A similar trend was observed for hGH expression. CSHP and AP media yielded 263 ± 49 µg/mL and 240 ± 29 µg/mL of hGH, respectively, whereas LB and VP media yielded 143 ± 20 µg/mL and 167 ± 21 µg/mL of hGH, respectively. Statistical analysis indicated no significant difference between CSHP and AP (*p* > 0.05) for either protein, demonstrating that CSHP performs equivalently to commercial AP in the production of costly therapeutic proteins. Although several studies have highlighted the suitability of fish-derived peptones in microbial fermentation, reports on their application in the production of therapeutic proteins (hSOD and hGH) remain limited. However, there is substantial evidence for extensive use of chum salmon by-products in cosmetics and pharmaceutical industries for its beneficial properties such as wound healing, angiogenesis promotion [54], anti-aging, anti-inflammatory effects [20] and neuroprotective applications [55]. Chum salmon-derived by-products are used in therapeutic protein production primarily as a source of collagen peptides [55] and polydeoxyribonucleotide (pDRN) [56], which are extracted from fish skin and sperm cells. The commercial success of salmon-derived ingredients validates the industrial viability of salmon by-product valorization for therapeutic purposes. In a previous study utilizing peptones derived from cutlassfish head, a 32% enhancement in hSOD expression was reported relative to the standard LB medium [13]. Furthermore, recombinant protein expressions were reduced in cultures supplemented with vegetable-based peptones relative to those supplemented with animal-derived peptones. This suggests that the nutrient composition of animal-based peptones, particularly those obtained from marine sources, may offer a more favorable amino acid profile for improved recombinant protein production. A scarcity of amino acids during protein translation can lead to growth arrest and a sudden halt in protein synthesis. Among these, cysteine, methionine, leucine, and alanine have been identified as important amino acids during protein production [57]. Therefore, a culture medium with a balanced amino acid profile is essential for optimal cell growth and productivity. Notably, CSHP contains a more balanced nutritional profile, with higher proportions of methionine and leucine compared to VP and AP (Table S7). Hence, integration of salmon processing waste into peptone production, we aimed to transform a low-value-by-product into a sustainable high value product.

### 3.4. Life Cycle Assessment-Based Quantitative Assessment of Environmental Benefits of Chum Salmon Head Peptone Production

LCA enables a comprehensive evaluation of the environmental friendliness of the materials by quantifying the cradle-to-grave carbon and greenhouse gas emissions. In the study, three raw materials, CSH, defatted soybean meal and skimmed milk, were processed using a standardized workflow, and all inputs/outputs were normalized to 1 kg peptone (dry basis) for comparability. Traditionally, peptone production has relied on casein derived from skim milk and, recently, on soybean hydrolysates. Hence, the ecoinvent LCA database used in this study lacked specific data for the enzymes used in traditional peptone processing. To address this, we represented them under the generic category “enzyme” in the LCA inventory. As the production scale and process efficiency are factors that significantly impact the results, we assumed identical energy usage for analogous processes for the LCA. To address representativeness, soybean- and skimmed milk–based peptone production was also recalculated using process parameters reported in patents and literature [28,29,30]. The detailed inventory data are provided in Table S3, and the comparative outcomes are illustrated in Figure S1. As expected, peptones extracted from CSH showed lower greenhouse gas emissions than those derived from skim milk or soybeans. Under identical conditions, production of CSHP resulted in 95.8% and 85.6% reductions in emissions compared with the production of skim milk- and soy-derived peptones, respectively (Table 4). For the LCA, all inventory data were normalized to a functional unit of 1 kg of peptone. This calculation was based on the observed 83.5% average recovery rate and the optimized 12% E/S ratio (0.6% enzyme at 5% substrate concentration), resulting in an actual enzyme input of 0.144 kg per kg of peptone. Notably, when recalculated using process parameters reported in patents and literature (Table S3), the comparative trend remained consistent: CSHP production still exhibited a 93% reduction in emissions compared with skimmed milk and a 79% reduction compared with soybean (Figure S1). The lower emissions associated with CSHP production may be attributed to the absence of methane emissions and respiration-related factors that are inherent to dairy cattle farming. To address concerns that burden-free treatment of fish heads as wastes might underestimate environmental burdens, we additionally conducted a sensitivity analysis under a co-product assumption using economic allocation (Table S4). This adjustment increased GWP by 8.1% (from 4.50 to 4.86 kg CO_2_-eq) for CSHP, yet the overall comparative advantage remained unchanged: marine-based peptones still show substantially lower climate impacts than conventional peptone sources.

## 4. Conclusions

The successful use of chum salmon head waste for enhancing recombinant protein production highlights an effective integration of sustainable resource utilization, waste reduction and biotechnological advancement. This approach also minimizes the environmental footprint associated with the culture media development. Thus, the current study represents a practical, cost-effective, and environmentally responsible strategy for advancing sustainable bioprocessing, aligning with the principles of the circular economy.

## Figures and Tables

**Table 1:** Weights of chum salmon fish heads, muscles, bones, remaining portions, and loss.

Chum Salmon Head Components	Wet Weight	Wet Weight (%)
Heads	10.4 kg	100%
Muscles	2.25 kg	21.6%
Bones	3.07 kg	29.6%
Remaining portions	3.08 kg	29.6%
Loss	2.00 kg	19.2%

Wet weight percentage (%) was calculated by comparing the weights of the flesh, bones, remaining portions, and loss to the total weight of the head.

**Table 2:** Molecular mass analysis of CSHP, AP, and VP.

Type of Peptone	Mn (Daltons)	Mm (Daltons)	Mp (Daltons)	PDI
CSHP	288	557	424	1.93
AP	487	1222	1361	2.51
VP	309	642	825	2.08

Mn, number average molecular mass; Mm, weight average molecular mass; Mp, molecular mass of highest peak; PDI, polydispersity index.

**Table 3:** Comparison of total nitrogen and amino-nitrogen contents of CSHP, AP, and VP.

Type of Peptone	Total Nitrogen (%)	Amino Nitrogen (%)
CSHP	12.5	4.9
AP	15.2	2.6
VP	11.5	3.4

CSHP, chum salmon head peptone; AP, animal peptone; VP, vegetable peptone.

**Table 4:** Comparison of environmental impact indicators in peptone production.

Impact Category	Product System		
	Chum Salmon Head (kg CO_2_-eq)	Skimmed Milk (kg CO_2_-eq)	Soybean (kg CO_2_-eq)
Biogenic GWP20	0.06 ± 0.08	78.7 ± 180	0.11 ± 0.15
Fossils + SLCFs GWP100	5.43 ± 3.38	88.9 ± 156	21.8 ± 24.7
Total incl. SLCFs GWP100	5.46 ± 3.40	131 ± 252	38.0 ± 51.0
Land use GTP50	0.01 ± 0.01	14.8 ± 33.8	14.8 ± 25.2

Fossil + SLCFs GWP100 represents fossil greenhouse gas emissions, including short-lived climate forces, expressed as 100-year global warming potential (GWP100). Total incl. SLCFs GWP100 represents total climate change impacts (fossil, biogenic and land-use CO_2_-eq), including short-lived climate forces.

## Data Availability

The data underlying this article are available in the article and in its online supplementary material.

## Supplementary Materials

The following supporting information can be downloaded at https://www.mdpi.com/article/10.3390/bioengineering13040409/s1. Figure S1. Greenhouse gas emissions of skimmed milk, soybean and CSHP production. GHG emissions are normalized to the functional unit of 1 kg peptone. CSHP showed a 93% reduction compared with skimmed milk and a 79% reduction compared with soybean. Table S1. Commercial enzymes used for CSH muscle hydrolysis. Table S2. Operating conditions for amino acid analysis. Table S3. Life cycle inventory for soybean- and skimmed-milk–based peptone production (1 kg peptone). Table S4. Sensitivity analysis of CSHP under allocation assumptions. Table S5. Life cycle inventory for CSHP (1 kg peptone). Table S6. Constituent and free amino acid contents of CSHP. Table S7. Relative composition of free amino acids in VP, AP, and CSHP. (The values were given as percentage form [%]).

## Abbreviations

The following abbreviations are used in this manuscript:

ANOVA	Analysis of variance
AP	Animal peptone
CSH	Chum salmon head
CSHP	Chum salmon head peptone
DNA	Deoxyribonucleic acid
GPC	Gel permeation chromatography
GWP100	Global warming potential
hGH	Human growth hormone
HPLC	High-pressure liquid chromatography
hSOD	Human superoxide dismutase
IPTG	Isopropyl-β-D-thiogalactopyranoside
LB	Luria–Bertani
PCR	Polymerase chain reaction
PDI	Polydispersity index
SDS-PAGE	Sodium dodecyl sulfate-polyacrylamide gel electrophoresis
VP	Vegetable peptone
