# Supplementary material for: Circular Approach to Biomanufacturing: Enhancing Therapeutic Protein Production Using Chum Salmon Head Peptone
Source: Bioengineering (Basel). 2026 Mar 31;13(4):409. doi: 10.3390/bioengineering13040409 (PMC13113008; doi:10.3390/bioengineering13040409)
Supplement: Supplementary file 1 [file bioengineering-13-00409-s001.zip › Table S7.pdf]

Table S7. Relative composition of free amino acids in VP, AP, and CSHP. (The values were given as percentage form [%])

Amino acid	VP	AP	CSHP
Aspartic acid	3.32	2.91	1.69
Glutamic acid	11.4	8.7	6.31
Asparagine	3.86	1.98	1.15
Serine	6.81	2.9	3.51
Glutamine	0.89	0.71	3.74
Histidine	2.57	1.73	5.05
Glycine	2.91	3.51	1.83
Threonine	5.69	2.99	3.36
Arginine	6.96	5.6	4.35
Alanine	7.4	7.13	6.85
Tyrosine	3.42	5.51	4.59
Valine	7.23	4.27	4.56
Methionine	2.76	1.36	6.45
Tryptophan	1.72	0.97	1.12
Phenylalanine	6.54	11.8	9.12
Isoleucine	6.25	4.22	4.49
Leucine	13.4	10.8	21.9
Lysine	5.16	21.7	8.15
Proline	1.83	1.31	1.82
