# Supplementary material for: Circular Approach to Biomanufacturing: Enhancing Therapeutic Protein Production Using Chum Salmon Head Peptone
Source: Bioengineering (Basel). 2026 Mar 31;13(4):409. doi: 10.3390/bioengineering13040409 (PMC13113008; doi:10.3390/bioengineering13040409)
Supplement: Supplementary file 1 [file bioengineering-13-00409-s001.zip › Figure S1.pdf]

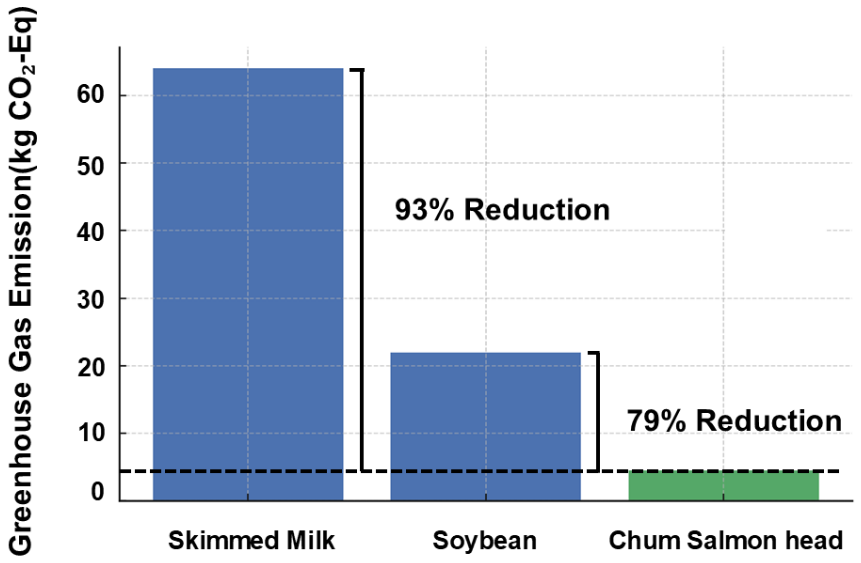

Figure S1. Greenhouse gas emissions of skimmed milk, soybean and CSHP production. GHG emissions are normalized to the functional unit of 1 kg peptone. CSHP showed a 93% reduction compared with skimmed milk and a 79% reduction compared with soybean.
