# Supplementary material for: Circular Approach to Biomanufacturing: Enhancing Therapeutic Protein Production Using Chum Salmon Head Peptone
Source: Bioengineering (Basel). 2026 Mar 31;13(4):409. doi: 10.3390/bioengineering13040409 (PMC13113008; doi:10.3390/bioengineering13040409)
Supplement: Supplementary file 1 [file bioengineering-13-00409-s001.zip › Table S1.pdf]

Table S1. Commercial enzymes used for CSH muscle hydrolysis.

**Enzyme**	**Activity**	**pH**
Bromelain from pineapple stem	>3 U/mg	6.0
Papain from papaya latex	1.5–10 U/mg	7.0
Pronase from *Streptomyces griseus*	7 U/mg	8.0
Protamex (proteases from *Bacillus* sp.)	>1.5 AU-N/g	7.0
Trypsin	>250000 USP units/g	8.0
