# Supplementary material for: Circular Approach to Biomanufacturing: Enhancing Therapeutic Protein Production Using Chum Salmon Head Peptone
Source: Bioengineering (Basel). 2026 Mar 31;13(4):409. doi: 10.3390/bioengineering13040409 (PMC13113008; doi:10.3390/bioengineering13040409)
Supplement: Supplementary file 1 [file bioengineering-13-00409-s001.zip › Table S2.pdf]

Table S2. Operating conditions for amino acid analysis

	**Amino acid**	**Free amino acid**
Instrument	Agilent 1200LC	Dionex Ultimate 3000
FL detector	Emission 450 nm, Excitation 340 nm (OPA)<br>Emission 305 nm, Excitation 266 nm (FMOC)	Emission 450nm, Excitation 340 nm (OPA)<br>Emission 305 nm, Excitation 266 nm (FMOC)
UV detector	338 nm	338 nm
Column	C18 column<br>(4.6 mm x 150 mm, 5 μm)	Inno C18 column<br>(4.6 mm x 150 mm, 5 μm)
Column temperature	40 ℃	40 ℃
Injection volume	0.5 μL	0.5 μL
Mobile phase	Mobile phase A:<br>ACN/MeOH/DW (9/9/1)<br>Mobile phase B:<br>40 mM Phosphate buffer<br>(pH 7.5)	Mobile phase A:<br>40 mM Sodium phosphate dibasic,<br>Mobile phase B: 3DW/ACN/MeOH<br>(10:45:45)<br>(pH 7)

OPA, o-phthalaldehyde; FMOC, 9-fluorenylmethyl chloroformate; DW, distilled water; ACN, acetonitrile; MeOH, methanol
