# Supplementary material for: Circular Approach to Biomanufacturing: Enhancing Therapeutic Protein Production Using Chum Salmon Head Peptone
Source: Bioengineering (Basel). 2026 Mar 31;13(4):409. doi: 10.3390/bioengineering13040409 (PMC13113008; doi:10.3390/bioengineering13040409)
Supplement: Supplementary file 1 [file bioengineering-13-00409-s001.zip › Table S3.pdf]

Table S3. Life cycle inventory for soybean- and skimmed-milk–based peptone production (1 kg peptone)

Step	Soybean peptone [26]	Skimmed-milk peptone [27, 28]
**1. Raw material preparation / Heat treatment**	**Input:**<br>Soybean (at plant): 5.7 kg<br>Electricity, medium voltage: 0.8 kWh<br>**Output:**<br>Protein: 5.5 kg<br>Residues: 0.2 kg	**Input:**<br>Skimmed milk (at plant): 33.0 kg<br>Electricity, medium voltage: 2.32 kWh **Output:**<br>Protein: 30 kg<br>Wastewater, untreated: 1.39 kg
**2. Hydrolysis / Isoelectric precipitation**	**Input:**<br>Protein: 5.5 kg<br>Enzyme (proxy): 0.03 kg<br>Tap water: 20.0 kg<br>Electricity: 3.5 kWh<br>**Output:**<br>Soluble protein: 25.53 kg	**Input:**<br>Protein: 30.0 kg<br>Hydrochloric acid: 0.5 kg<br>Electricity: 0.25 kWh<br>**Output:**<br>Protein: 29.0 kg<br>Residues: 0.5 kg<br>Wastewater, untreated: 1.5 kg
**3. Enzyme inactivation / Centrifugation**	**Input:**<br>Protein solution: 25.53 kg<br>Electricity: 1.48 kWh<br>**Output:**<br>Protein: 25.53 kg	**Input:**<br>Protein: 29.0 kg<br>Electricity: 1.8 kWh<br>**Output:**<br>Protein: 25.0 kg<br>Solid residues: 5.0 kg
**4. Filtration & concentration / Fractionation**	**Input:**<br>Protein: 25.53 kg<br>Electricity: 3.0 kWh<br>**Output:**<br>Protein concentrate: 2.73 kg<br>Residues: 3.8 kg<br>Wastewater, untreated: 19 kg	**Input:**<br>Protein: 25.0 kg<br>Ammonium sulfate: 9.8 kg<br>Electricity: 0.1 kWh<br>**Output:**<br>Protein: 21.5 kg<br>Residues (salt/biomass): 13.3 kg
**5. Drying / Second centrifugation**	**Input:**<br>Protein: 2.73 kg<br>Electricity: 5.2 kWh<br>**Output:**<br>Peptone (final product): 1 kg	**Input:**<br>Protein: 21.5 kg<br>Electricity: 1.29 kWh<br>**Output:**<br>Protein: 18.0 kg<br>Residues: 3.5 kg
**6. — / Dialysis**	—	**Input:**<br>Protein: 18.0 kg<br>Sodium phosphate: 0.12 kg<br>Electricity: 0.4 kWh<br>**Output:**<br>Protein: 5.0 kg<br>Residues: 2.5 kg<br>Wastewater, untreated: 10.62 kg
**7. — / Freeze-drying**	—	**Input:**<br>Protein: 5.0 kg<br>Electricity: 4.0 kWh<br>**Output:**<br>Peptone (final product): 1 kg<br>Residues: 4 kg

Stepwise mass and energy inputs/outputs for soybean- and skimmed milk–based peptone production (based on published patents and literature), expressed per functional unit of 1 kg peptone.
