# Supplementary material for: Circular Approach to Biomanufacturing: Enhancing Therapeutic Protein Production Using Chum Salmon Head Peptone
Source: Bioengineering (Basel). 2026 Mar 31;13(4):409. doi: 10.3390/bioengineering13040409 (PMC13113008; doi:10.3390/bioengineering13040409)
Supplement: Supplementary file 1 [file bioengineering-13-00409-s001.zip › Table S4.pdf]

Table S4. Sensitivity analysis of CSHP under allocation assumptions

System	Method	Peptone		Low-value co-product
**Chum Salmon head**	Physical	78.4%		21.6%
	Economic	92.5%		7.50%
**Impact category**	**Unit**	**Burden-free**	**Co-product**	**Change (%)**
**GWP (100 yr)**	kg CO_2-eq	4.50	4.86	+8.1%

Allocation results for salmon head coproduct systems. Physical allocation is based on output mass fractions of fillet versus head, derived from FAO Technical Papers on Fish Processing Yields (2020–2022). Economic allocation is based on product revenues (mass × unit price), using fillet prices (6–8 USD/kg) from Korea Maritime Institute (KMI) Fishery Outlook Reports (2022–2023) and FAO Globefish Market Reports, and head prices for fishmeal/by-product use (0.3–0.5 USD/kg) from Korea Rural Economic Institute (KREI) by-product statistics cross-checked with FAO Globefish data. Currency values were normalized to 2022 USD using Bank of Korea annual average exchange rates. Greenhouse gas emissions (GWP100) were calculated following ISO 14044 allocation principles. Applying economic allocation increased GWP by 8.1% (from 4.50 to 4.86 kg CO_2-eq), but this adjustment does not alter the overall conclusion that salmon head–based peptone remains substantially less impactful than land-based systems (soy and milk peptones).
