# Supplementary material for: Circular Approach to Biomanufacturing: Enhancing Therapeutic Protein Production Using Chum Salmon Head Peptone
Source: Bioengineering (Basel). 2026 Mar 31;13(4):409. doi: 10.3390/bioengineering13040409 (PMC13113008; doi:10.3390/bioengineering13040409)
Supplement: Supplementary file 1 [file bioengineering-13-00409-s001.zip › Table S5.pdf]

Table S5. Life cycle inventory for CSHP (1 kg peptone)

Step	Process	Inputs	Operating conditions	Outputs
1	Steaming	Chum salmon head: 10.4 kg (wet weight)<br>Tap water: 12.0 kg<br>Heat, from natural gas (>100 kW): 3.8 MJ	100 °C, 30 min; steaming efficiency 89.7%	Cooked biomass (muscle fraction separated)<br>Wastewater: 12.0 kg
**2**	Raw material preparation	Muscle fraction from salmon head<br>Electricity: 2.0 kWh (freeze-drying + grinding)	Freeze-drying, grinding	Muscle powder (used for hydrolysis)
**3**	Enzymatic hydrolysis	Muscle powder slurry: 1.2 kg (protein 20.2%)<br>Enzyme: 0.144 kg<br>Tap water: 24.0 kg<br>Electricity: 0.5 kWh	55 °C, 6 h; pH 7.0; substrate 5% w/v; E/S ≈ 0.12 g/g	Hydrolysate (requires inactivation)<br>Wastewater (dilution losses)
**4**	Enzyme inactivation	Heat, from natural gas (>100 kW): 0.41 MJ	95 °C, 10 min	Enzyme fully inactivated
**5**	Centrifugation	Hydrolysate<br>Electricity: ~1.0 kWh	10,000 rpm, 20 min	Supernatant (soluble fraction)<br>Pellet (insoluble residues)
**6**	Freeze-drying	Soluble fraction<br>Electricity: 4.78 kWh	−55 °C, 50 h; vacuum 8.87	Peptone (product): 1 kg<br>Water, evaporated: 3.2 kg

Stepwise mass and energy inputs/outputs for CSHP production, expressed per functional unit of 1 kg peptone.
