# Supplementary material for: Circular Approach to Biomanufacturing: Enhancing Therapeutic Protein Production Using Chum Salmon Head Peptone
Source: Bioengineering (Basel). 2026 Mar 31;13(4):409. doi: 10.3390/bioengineering13040409 (PMC13113008; doi:10.3390/bioengineering13040409)
Supplement: Supplementary file 1 [file bioengineering-13-00409-s001.zip › Table S6.pdf]

Table S6. Constituent and free amino acid contents of CSHP.

Amino acid	Constituent amino acids (g/kg)	Constituent amino acids (%)	Free amino acids (g/kg)	Free amino acids (%)
Aspartic acid	74.6	10.4	1.14	1.48
Glutamic acid	128	17.9	4.27	5.55
Asparagine	n. a.*	n. a.	0.78	1.01
Serine	32.0	4.46	2.38	3.09
Glutamine	n. a.	n. a.	2.53	3.29
Histidine	19.8	2.76	3.42	4.44
Glycine	45.8	6.39	1.24	1.61
Threonine	37.6	5.24	2.27	2.95
Arginine	45.8	6.38	2.94	3.82
Alanine	48.9	6.83	4.64	6.03
Tyrosine	22.6	3.15	3.10	4.03
Valine	38.8	5.42	3.09	4.01
Methionine	n. a.	n. a.	4.37	5.67
Tryptophan	n. a.	n. a.	0.76	0.99
Phenylalanine	29.0	4.04	6.17	8.01
Isoleucine	35.1	4.90	3.04	3.95
Leucine	58.6	8.17	14.8	19.2
Lysine	63.3	8.83	5.51	7.16
Proline	29.9	4.16	1.23	1.60
Citrulline	0.50	0.07	0.35	0.45
Taurine	6.62	0.92	8.96	11.6

Total	717	100	77.0	100

*n. a., Not available
